# Social (pragmatic) communication disorder: a research review of this new DSM-5 diagnostic category

**DOI:** 10.1186/1866-1955-6-41

**Published:** 2014-11-27

**Authors:** Lauren B Swineford, Audrey Thurm, Gillian Baird, Amy M Wetherby, Susan Swedo

**Affiliations:** Pediatrics and Developmental Neuroscience Branch, National Institute of Mental Health, 10 Center Drive MSC 1255, Building 10, Room 1C250, Bethesda, MD 20892-1255 USA; Guy’s and St Thomas’ NHS Foundation Trust, King’s Health Partners Paediatric Neurosciences St Thomas Hospital, London, SE1 5HE UK; Department of Clinical Science, Florida State University, 1940 North Monroe Street, Suite 72, Tallahassee, FL 32303 USA

**Keywords:** Social communication disorder, Pragmatic language impairment, Autism spectrum disorder, DSM-5

## Abstract

Social (pragmatic) communication disorder (SCD) is a new diagnostic category in the Diagnostic and Statistical Manual of Mental Disorders, fifth edition (DSM-5). The purpose of this review is to describe and synthesize the relevant literature from language and autism spectrum disorder (ASD) research relating to pragmatic language impairment and other previously used terms that relate to SCD. The long-standing debate regarding how social communication/pragmatic impairments overlap and/or differ from language impairments, ASD, and other neurodevelopmental disorders is examined. The possible impact of the addition of SCD diagnostic category and directions for future research are also discussed.

## Introduction

Social (pragmatic) communication disorder (SCD) is a new diagnostic category included under Communication Disorders in the Neurodevelopmental Disorders section of the Diagnostic and Statistical Manual of Mental Disorders, fifth edition (DSM-5) [1]. SCD is defined by a primary deficit in the social use of nonverbal and verbal communication (see Table 1 for the full list of criteria). Individuals with SCD may be characterized by difficulty in using language for social purposes, appropriately matching communication to the social context, following rules of the communication context (e.g., back and forth of conversation), understanding nonliteral language (e.g., jokes, idioms, metaphors), and integrating language with nonverbal communicative behaviors. Sufficient language skills must be developed before these higher-order pragmatic deficits can be detected, so a diagnosis of SCD should not be made until children are 4–5 years of age. Social communication disorder can co-occur with other communication disorders in the DSM-5 (these include language disorder, speech sound disorder, childhood-onset fluency disorder, and unspecified communication disorder), but cannot be diagnosed in the presence of autism spectrum disorder (ASD) [1].

**Table 1:** **Social (pragmatic) communication disorder**

Diagnostic Criteria 315.39 (F80.89)	
A. Persistent difficulties in the social use of verbal and nonverbal communication as manifested by all of the following:	
	1. Deficits in using communication for social purposes, such as greeting and sharing information, in a manner that is appropriate for the social context.
	2. Impairment of the ability to change communication to match context or the needs of the listener, such as speaking differently in a classroom than on a playground, talking differently to a child than to an adult, and avoiding use of overly formal language.
	3. Difficulties following rules for conversation and storytelling, such as taking turns in conversation, rephrasing when misunderstood, and knowing how to use verbal and nonverbal signals to regulate interaction.
	4. Difficulties understanding what is not explicitly stated (e.g., making inferences) and nonliteral or ambiguous language (e.g., idioms, humor, metaphors, multiple meanings that depend on the context for interpretation).
B. The deficits result in functional limitations in effective communication, social participation, social relationships, academic achievement, or occupational performance, individually or in combination.	
C. The onset of symptoms is in the early developmental period (but deficits may not become fully manifest until social communication demands exceed limited capacities).	
D. The symptoms are not attributable to another medical or neurological condition or to low abilities in the domains of word structure and grammar, and are not better explained by autism spectrum disorder, intellectual disability (intellectual developmental disorder), global developmental delay, or another mental disorder.	

Reprinted with permission from the Diagnostic and Statistical Manual of Mental Disorders, Fifth Edition, (Copyright 2013). American Psychiatric Association.

The origins of SCD are in the speech and language literature, which documents pragmatic language impairment as a distinct pattern of deficits in social use of language. It is worth noting that the DSM-5 SCD criteria explicitly include nonverbal communication, while traditionally, pragmatic language did not. Still, the terms social communication and pragmatic impairments are often used interchangeably in the literature, so it is perhaps unsurprising that individuals with these primary deficits have also been described frequently in neuropsychological literature [2, 3] and ASD literature [4, 5].

In the DSM-5, ASD is a new diagnostic category in the Neurodevelopmental Disorders section, characterized by impairments in social communication and social reciprocity and by the presence of restricted interests and repetitive behaviors. DSM-5 ASD replaces the disorders that comprised the DSM-IV pervasive developmental disorders (PDD) category, including autistic disorder, Asperger’s disorder, and pervasive developmental disorder, not otherwise specified (PDD-NOS).

The inclusion of SCD was partially driven by the transition from DSM-IV PDD to DSM-5 ASD and the subsequent loss of DSM-IV PDD-NOS. PDD-NOS was a broad diagnostic category that included all conditions in which “there is severe and pervasive impairment in the development of reciprocal social interaction associated with impairment in either verbal or nonverbal communication skills or with the presence of stereotyped behaviors, interests, and activities” [6]. Because DSM-5 ASD criteria require the presence of repetitive behaviors, some have raised the concern that some individuals who met the DSM-IV PDD-NOS criteria no longer have a diagnostic home and will therefore be ineligible for the treatment services appropriate for their impairments. Thus, SCD and ASD are common in the requirement of deficits in social communication skills, but individuals with SCD cannot evidence restricted interests, repetitive behaviors, insistence on sameness, or sensory abnormalities. It is essential to rule out a diagnosis of ASD by verifying the lack of these additional symptoms, currently or by history, before assigning a diagnosis of SCD. Thus, the criteria for SCD are qualitatively different from ASD and are not equivalent to “mild ASD.” However, whether children display the specific pattern of the SCD diagnostic criteria is still an empirical question, as the criteria have just been added to the DSM and children with severe social communication difficulties and without significant repetitive behaviors are often overlooked in the literature [7].

Although SCD may serve as a diagnostic home for individuals who would have previously met the criteria for DSM-IV PDD-NOS, the goal of new DSM diagnostic categories is not to prevent the loss of previously diagnosed disorders, but instead to represent natural phenomena as accurately as possible. Therefore, the rationale for the addition of SCD to DSM-5 communication disorders was rooted in literature suggesting that the impairment in pragmatics that is observed in individuals with significant social communication deficits can be differentiated from the structural and formulation difficulties that characterize language disorder. However, a long-standing debate exists regarding the nature of the overlap between social communication/pragmatic impairments and other communication and neurodevelopmental disorders. The introduction of the SCD diagnosis does not settle this debate, but instead gives researchers a tool with which to develop empirical evidence to answer the question.

Thus, in addition to the question of the overlap between SCD and other language disorders, important questions exist surrounding the practice of ruling out DSM-5 ASD when SCD is diagnosed [8]. Since the publication of the DSM-5, there has been a focus on how these particular issues may affect clinical practice [8]. While such discussions are important, the current review focuses on previous research relating to the new DSM-5 category and outlines research to be conducted to investigate the validity of the new diagnostic category. As described below, SCD aligns conceptually and practically with pragmatic language impairment, although SCD was purposefully expanded to incorporate difficulties with nonverbal communication. From the existing literature, we summarize what is known about differential diagnosis, familial aggregation, developmental course, and prognosis. We also discuss the possible impact of changes in the DSM-5 ASD criteria. We emphasize here and throughout that given the variable definitions used for pragmatic language impairment in the past and the broader definition of SCD in the DSM-5, it is not yet known if and how the extant literature on pragmatic language impairment will relate to findings for SCD.

## Review

### Distinguishing pragmatic impairments from language disorders

In the early 1980s, Rapin and Allen [9] introduced the term semantic-pragmatic deficit syndrome to characterize children who are overly verbose, demonstrate word finding difficulty, and have difficulty with conversation including poor topic maintenance. Similarly, Bishop and Rosenbloom [10] used the term semantic-pragmatic disorder to describe children who have difficulty understanding and following the rules of conversation and may use unusual language or word choice to communicate. However, it has been suggested that semantic deficits may not always co-occur with pragmatic deficits. To differentiate individuals with deficits in pragmatics (though not necessarily semantics), the term pragmatic language impairment was coined [11]. Further research has confirmed that the clinical characteristics of pragmatic language impairment include difficulties understanding and using language in context and/or following the social rules of language, despite relative strengths in word knowledge and grammar [12, 13]. However, the boundaries of pragmatic impairments have not been consistently defined in the literature. Specifically, there has been considerable debate about whether pragmatic language impairment can be fully separated from the social and communication impairments of other language disorders [14].

Pragmatic language impairments have been described most often among children with what is referred to in the literature as specific language impairments. Specific language impairments are characterized by delays in language skills in the absence of other developmental delays. While the term specific language impairment has not been used in the DSM [15], it is a term widely used in research and has been used extensively by speech and language pathologists [16]. In previous versions of the DSM, characteristics of specific language impairments were reflected in the criteria for expressive language disorder and mixed receptive expressive language disorder, which are now combined as language disorder in DSM-5.

Empirical evidence of pragmatic language impairment comes from studies using standardized measures of language abilities as well as teacher report to describe six subgroups of language impairment, including children with primary deficits in (1) syntax/morphology and receptive language; (2) phonology, expressive language, and poor word reading; (3) articulation, phonology, and expressive language; (4) articulation, phonology, and expressive language with higher profiles than group 3; (5) articulation, phonology, and syntax/morphology with expressive and/or receptive difficulties; and (6) semantics and/or pragmatics. Thus, one subgroup presented primarily with semantic-pragmatic deficits, as described above, although it was identified mainly by teacher report, since standardized tests were poor at detecting these impairments [12, 17]. However, it is possible that this subgroup with primary deficits in pragmatics overlaps with individuals with deficits in structural language (form, content) as some children with structural language impairments also had significant impairments in pragmatic language [18]. There is also evidence to suggest that pragmatic language impairments constitute a fundamentally different form of communication disorder, as some children have been found to demonstrate primary deficits in using language rather than the structure of language [19].

### Distinguishing pragmatic impairments from ASD

Given that impairments in social communication are a hallmark feature of ASD, overlap in the symptomatology of SCD and ASD is expected. Specifically, children with ASD who have adequate structural language abilities may have pragmatic difficulties such as verbosity, overly formal speech, and trouble taking turns in conversation. These pragmatic deficits are expressed in the context of a larger constellation of ASD symptoms, which include impairments in social reciprocity and the presence of restricted interests and repetitive behaviors. On the other hand, it is important to note that pragmatic language deficits have been reported to occur without the impairing social deficits and repetitive behaviors indicative of ASD, suggesting that two distinct patterns of symptoms exist [5, 9, 20]. These diagnostic subtleties between ASD (which includes pragmatic impairments) and isolated pragmatic impairments are reflected in a review by Brook and Bowler [14]. Specifically, these authors report that children described as having semantic-pragmatic impairments have similar deficits to children reported in the autism literature and conclude that further research is needed to understand any underlying differences between ASD and semantic-pragmatic impairments. Thus, it is essential for research to be conducted to develop a systematic method of measuring and documenting the distinctness (or lack thereof) of ASD and SCD. This method will need to include comprehensive assessment of current behavior as well as past history.

It is possible that children with pragmatic language impairment fall along a continuum between children with specific language impairment and those with social communication deficits of ASD [20]. The DSM-IV diagnostic criteria for PDD-NOS required impairment in reciprocal social interaction and either impairment in communication skills or the presence of stereotyped behavior, interests, and activities. The PDD-NOS category also allowed subclinical symptoms. Thus, it is likely that individuals with social communication and/or pragmatic language impairment were assigned a diagnosis of DSM-IV PDD-NOS. In fact, one study found that all but two of 66 children (mean age 9 years) diagnosed with PDD-NOS had “one distinct symptom pattern, namely social impairments without significant repetitive and stereotyped behaviors” [4]. These findings are not surprising given that the DSM-IV PDD diagnostic criteria did not distinguish language impairments from deficits in social communication.

Other extant studies that investigated more broadly defined pragmatic language impairments have reported that significant proportions of individuals with pragmatic language impairments did not meet the DSM-IV criteria for autistic disorder [21, 22]. It is less clear whether such individuals will meet the DSM-5 ASD criteria. For example, the inclusion of patient history rather than only present symptoms in the DSM-5 criteria for ASD may mean that some individuals who currently have only deficits of social communication would still receive an ASD diagnosis because they had a history of repetitive behavior/restricted interests. Importantly, stereotyped language is now part of the repetitive and restrictive behavior domain for ASD. Thus, stereotyped language, which is considered part of the clinical profile of individuals with pragmatic language impairment [20], would now also count as one of the two required symptoms of repetitive and restrictive behavior for an ASD diagnosis. Given the mutual exclusivity of ASD and SCD, the presence of stereotyped language in the restrictive and repetitive behavior domain may have significant impact on who is considered for a possible diagnosis of SCD.

### Pragmatic impairments and other developmental or behavior problems

Pragmatic language difficulties have been described in a variety of psychiatric and neurodevelopmental disorders, including schizophrenia [23], bipolar disorder [24], and attention deficit hyperactivity disorder [25], among others. With respect to attention deficit hyperactivity disorder, it has been hypothesized that the primary symptoms of the disorder (i.e., impulsiveness, inattention, hyperactivity) may cause impairments in social communication, which result in additional limitations on communication, social participation, and academic achievement [2, 26]. Pragmatic impairments have also been found frequently among children with neurologic conditions, such as epilepsy [27] and among children with behavioral problems [13, 28]. Additional research is needed to understand the impact that SCD and pragmatic language impairments have on the acquisition of academic skills, problematic behaviors, and neuropsychiatric disorders.

### Familial aggregation and genetics

While there is no published research regarding the heredity and/or genetics of SCD, there has been some research showing familial aggregation of social communication difficulties (albeit mostly in the context of autism symptom heritability) and a burgeoning literature of studies exploring genetic associations. With respect to familial aggregation, pragmatic language difficulties have been found to run in families of children with autism [29–31] as well as in families of individuals with specific language impairment [32]. Moreover, pragmatic language (as well as structural language) has been found to be more impaired among probands with ASD whose parents are categorized as broader autism phenotype, when compared to those whose parents are not [33]. Further, studies have shown significant heritability of pragmatic skills in families that include individuals with both autism and specific language impairment, indicating nonadditive genetic effects [34].

Thus far, there has been limited study of genes associated with isolated pragmatic language impairments or social communication traits. Studies are starting to report associations of pragmatic language impairments with common variants of specific genes [35, 36]. However, a recent study that measured social communication traits over time found that there were developmental changes in the strength of genetic effects [37], highlighting the need to also investigate the influence of environmental effects. Also, other studies have found that genes associated with pragmatic language impairments were nonspecific and were also found in association with neurocognitive deficits, intellectual disability, ASD, other psychiatric disorders, and other language impairments [38, 39]. This lack of specificity highlights the tension between categorizing based on specific symptoms that require specific treatment, while at the same time indicating common etiologies for several communication and neurodevelopmental disorders.

In contrast to this lack of specificity, studies have begun to find genetic association distinctions between structural and pragmatic language. For instance, a study that focused on duplications in 7q11.23 found that this genetic abnormality was associated with significant speech impairments, with relative strengths in pragmatics [40]. Interestingly, research on supernumerary X and Y chromosomes has also contributed to findings in this area. A recent study found that while additions of both X and Y chromosomes were related to both structural and pragmatic impairments on a rating scale, additions of a Y chromosome were specifically related to pragmatic language impairment [41].

### Course and prognosis of SCD

At this point, it is impossible to discuss the course and prognosis of SCD. The paucity of longitudinal research limits our attempts to extrapolate from previous literature to SCD. While one study found that a significant minority of school-aged children continued to demonstrate pragmatic impairments for at least a year, some children improve their pragmatic skills but continue to have other types of language problems [12]. The only study examining longer-term stability suggests that pragmatic language impairment may be rather stable into adulthood but the sample size was insufficient to generalize the findings [42].

Research has begun to explore the long-term psychosocial outcomes of children with pragmatic language impairment. When compared to peers with specific language impairment, one longitudinal study suggested that children with pragmatic language impairment generally perform better in academics, but have persistent pragmatic difficulties [42]. The pragmatic difficulties seen in adulthood appeared to most negatively impact the ability to have close friendships or romantic relationships. In addition, when compared to a control group of adults with ASD, adults with pragmatic language impairment and specific language impairment showed significantly fewer behaviors characteristic of ASD, providing support for a diagnostic distinction between pragmatic language impairment and ASD.

### Diagnostic and change measures of pragmatic language

Table 2 presents measures currently available to assess pragmatic language, which may serve as a starting point for quantification of SCD, although they may not all be applicable to all of the criteria required for a diagnosis of SCD. As noted below, psychometric information is limited for most assessment tools available. Two standardized measures available include the Test of Pragmatic Language [43] and the Comprehensive Assessment of Spoken Language [44]. The Test of Pragmatic Language is a norm-referenced measure for children between 6 and 18 years of age which measures several aspects of pragmatic communication including physical setting, audience, topic, purpose, visual gestural cues, and abstraction. The Comprehensive Assessment of Spoken Language is an omnibus test of expressive language including four subtests to measure pragmatics for individuals between 3 and 21 years of age. Specific areas of pragmatics measured on the Comprehensive Assessment of Spoken Language include pragmatic judgment, idiomatic language, nonliteral language, and inferencing. While both of these standardized measures capture aspects of pragmatic language that are diagnostic features of SCD, the dichotomous scoring systems (i.e., correct or incorrect) make it difficult to measure the quality of pragmatic language.

**Table 2:** **Measures of pragmatic abilities**

Test name and author	Age range	Norm referenced	Measures/subtests	Categorization/cutoffs
Direct assessment				
Test of Pragmatic Language (Phelps-Terasaki and Phelps-Gunn, 1992) [43]	6:0–18:11	Yes	Measures six core subcomponents of pragmatics: physical setting, audience, topic, purpose (speech acts), visual-gestural cues, and abstraction	This test provides quotients, percentile ranks, and age equivalents, calculated at each 6-month interval. The summary score, called the Language Quotient, is expressed as a standard score with a mean of 100 (SD = 15). A cutoff score of 79 was chosen as indicating pragmatic impairment
Pragmatic Rating Scale (Landa, 1992) [30]	9:0 and above	No	Identifies 19 pragmatic behaviors. Ratings are based on conversational behavior observed throughout the session, including a 15-min conversation (during ADOS)	Each pragmatic behavior is rated on a three point scale, with 0 indicating normal behavior, 1 indicating moderately abnormal behavior not considerably disruptive to the conversation, and 2 indicating that the behavior was strikingly abnormal
Comprehensive Assessment of Spoken Language (Carrow-Woolfolk, 1999) [44]	3:0–21:0	Yes	An omnibus test of general verbal language. Four subtests are designed to assess pragmatics: pragmatic judgment, idiomatic language, nonliteral language, and inference	Provides standard scores (M = 100, SD = 15), age equivalents, and percentiles
Rating scales				
Children’s Communication Checklist (Bishop, 2003) [47]	4:0–16:11	Yes	70-item questionnaire that measures structural language (speech, syntax, semantics, and coherence) and pragmatic language (initiation, scripted, context, nonverbal communication, social relations, and interests)	The five pragmatic scales can be combined into a pragmatic composite. A pragmatic composite score ≤132 best identified children with pragmatic language impairment
The Pragmatics Profile of Everyday Communication Skills in Children (Dewart and Summers, 1995) [48]	Preschool version: birth to 4:0. School-age version: 5:0–10:0	No	Interview that measures four areas of pragmatics: communicative function, response to communication, interaction and conversation, and contextual variation	Provides descriptive information only used to identify strengths and weaknesses and to develop treatment goals
Language Use Inventory (O’Neill, 2002) [49]	1:6–3:11	Yes	Fourteen subscales assessing communication with gestures, words, and longer sentences for a variety of functions	Provides percentile ranks for 1-month age bands
Pragmatic Protocol (Prutting and Kirchner, 1987) [46]	5:0 and above	No	Rating scale completed after observing spontaneous and unstructured conversation which measures verbal, nonverbal, and paralinguistic aspects of pragmatic language	Provides descriptive information (appropriate, inappropriate, or no opportunity to observe) for 30 items

The Pragmatic Rating Scale [30] is completed based on conversational behavior observed throughout a session, including a 15-min conversation typically drawn from the Autism Diagnostic Observation Schedule [45]. The Pragmatic Rating Scale measures 19 pragmatic behaviors, (all of which are part of the SCD criteria), which are scored on a three-point scale ranging from normal behavior to strikingly abnormal behavior. The Pragmatic Protocol [46] which is used for children aged 5 and above also is completed after observing a child in a 15-min unstructured conversation. This measure uses a categorical rating (i.e., appropriate, inappropriate, no opportunity) to score several aspects of verbal behavior (i.e., speech acts, topics, turn-taking, lexical selection, stylistic variations), paralinguistic aspects (i.e., intelligibility and prosody), and nonverbal aspects (i.e., kinesics and proxemics). While both measure pragmatic skills relevant to SCD, neither are norm-referenced nor is information available regarding their validity or reliability.

Parent and teacher rating scales are often used to gather information regarding a child’s pragmatic abilities. The most commonly used rating scale within research settings is the Children’s Communication Checklist, second edition [47]. This edition of the Children’s Communication Checklist is a 70-item parent questionnaire for children between 4 and 16 years of age designed to measure pragmatic abilities in the context of their overall language skills. It has 10 subscales and provides scores for two composites: a general communication composite and a social-interaction deviance composite to identify children whose pragmatic deficits are significant compared to their structural language skills. The second edition of the Children’s Communication Checklist is one of the few norm-referenced and validated questionnaires to measure pragmatic deficits [48].

Also available is the Pragmatics Profile of Everyday Communication Skills in Children [49] which measures communicative function, responses to communication, interaction and conversation, and contextual variation. There are also measures of pragmatic language available for children younger than 4 years, such as the Language Use Inventory [50], which may be more useful in predicting later SCD than in diagnosing, since it does not include comprehensive questions pertaining to higher-level language skills such as conversation or inferencing. Recent research supports the reliability and predictive validity of this tool [51, 52].

## Conclusions

Extrapolating from research descriptions of pragmatic language impairment, an unknown percentage of children are expected to exhibit significant pragmatic communication difficulties. Evidence of SCD was provided in the DSM-5 field trials, which indicated that a decrease in DSM-IV ASD diagnoses was accounted for by movement to SCD diagnostic category [53]. The difficulties that define SCD can include, but are not limited to, one’s ability to have back-and-forth conversations and understand and express nuances in communication contexts and implicit language. The inclusion of SCD in the DSM-5 gives impetus to extend what is known regarding social (pragmatic) communication disorders using the operationalized diagnostic criteria, and to systematically move the field forward to better understand and document the essential characteristics and validity of SCD. Further, the addition of SCD as a diagnostic category in DSM-5 will help individuals with these symptoms access appropriate care that is tailored to addressing these symptoms. The use of the SCD diagnosis in research will help to document the effectiveness of targeted treatments, to determine needed treatment intensity to impact symptoms, and to identify individual symptom response to specific treatments.

An important next step will be to examine the validity of the SCD criteria. Because no biological markers and few definitive objective measures exist for SCD, the gold standard in diagnosis must utilize the combination of clinical skills and expertise along with standardized testing [54]. Accordingly, it will be critical that researchers include a thorough developmental history along with cognitive, language, and ASD testing to rule out a diagnosis of ASD and adequately characterize the individuals affected and research samples obtained. Further, five criteria have been proposed for establishing validity of psychiatric diagnoses which could be applied to neurodevelopmental disorders and include: 1) clinical description (clinical and socioeconomic features), 2) laboratory studies (behavioral and biological), 3) delimitation from other disorders (specify exclusion criteria), 4) follow-up studies (course of illness and diagnostic stability), and 5) family studies [55]. Once samples of individuals with SCD are obtained, the diagnostic validity of the assessments used needs to be examined. The diagnostic criteria for SCD suggest that the symptoms exist along one dimension and this conceptualization of impairments needs to be empirically tested to demonstrate construct validity.

Future studies should evaluate the SCD criteria before attempts are made to measure the prevalence of SCD. Previous methods for estimating prevalence of ASD such as reviewing evaluation records to look for the presence of the diagnostic criteria [56] are likely to be insufficient for SCD. In-person evaluations may be required, due to the nature of the SCD clinical presentation. Thus, this will be a large task that requires multiple investigators and a significant investment of resources. Through estimating the prevalence of SCD, researchers would also be able to evaluate the impact of socio-demographic and cultural factors on its occurrence.

To address continued questions, future research should also examine areas of overlap between SCD and the DSM-5 criteria for language disorder. Comorbidity of SCD with other communication disorders is permitted so the frequency of overlap in these diagnoses will be informative with respect to how separable pragmatic language problems are from reduced vocabulary and limited sentence structure. Thus, comprehensive language testing should be included in the diagnostic assessment of SCD to characterize not only the pragmatic aspects of communication but also grammatical and semantic aspects. Given that the DSM-5 SCD criteria expand upon pragmatic language impairment by including nonverbal communication, further study of the validity of the diagnosis is needed. Research examining if and how the social communication deficits of SCD differ from those in ASD qualitatively or quantitatively is also needed.

Also needed are longitudinal studies of the course and stability of social communication and pragmatic deficits across development. Again, to consider a diagnosis of SCD, children must possess adequate speech and language abilities (i.e., emerging by 4–5 years of age in typical language development) to detect specific verbal pragmatic deficits. Thus, research should focus on samples of preschool- and school-aged children as a baseline of symptom presentation. Also, to understand the course of the disorder, research samples will need to be followed longitudinally. Longitudinal samples will allow for factor analytic examination of the diagnostic criteria while controlling for age and other factors such as developmental level. One step towards measuring the course of the disorder will be the development and/or validation of assessment tools to measure and track diagnostic features of SCD and change over time.

In sum, although SCD is a new, untested entity, clinicians and investigators can learn much from existing literature on pragmatic language impairment and other neurodevelopmental pragmatic impairments. The ultimate goals will be refinement of conceptualization, development and validation of assessment tools and interventions, and comprehensive understanding of shared and unique etiologic factors for SCD in relation to other neurodevelopmental disorders.

## Authors’ information

LS, AT, and SS are with the Pediatrics and Developmental Neuroscience Branch, National Institute of Mental Health. GB is with Guy’s and St Thomas’ NHS Foundation Trust, London, UK. AW is with Department of Clinical Science, Florida State University.

## Abbreviations

ADOS: Autism Diagnostic Observation Schedule—general
ASD: autism spectrum disorder
DSM-IV: Diagnostic and Statistical Manual of Mental Disorders, fourth edition
DSM-5: Diagnostic and Statistical Manual of Mental Disorders, fifth edition
PDD-NOS: pervasive developmental disorder, not otherwise specified
SCD: social (pragmatic) communication disorder.
